# Pharmacists in general practice: what do they do? A qualitative case study

**DOI:** 10.1007/s11096-023-01619-4

**Published:** 2023-08-12

**Authors:** Thomas Gerardus Hendrik Kempen, Rouzi Koumi, Sofia Kälvemark Sporrong

**Affiliations:** 1 Department of Pharmacy, Uppsala University, Uppsala, Sweden; 2 Division of Pharmacoepidemiology and Clinical Pharmacology, Utrecht Institute for Pharmaceutical Sciences, Utrecht University, Utrecht, The Netherlands; 3 Nivel, Netherlands Institute for Health Services Research, Utrecht, The Netherlands; 4 Apoteket S:ta Ragnhild, Södertälje, Sweden

**Keywords:** Case study, Drug-related problems, General practice, Medication review, Pharmacists, Scope of practice, Task performance

## Abstract

**Background:**

Pharmacists working in general practice are a relatively new phenomenon in many European countries. Providing insight into what pharmacists do in general practice may support further implementation of general practice-based pharmacist roles and enhance their contribution to health care.

**Aim:**

To explore the tasks performed by pharmacists in general practice in Sweden.

**Method:**

A case study was conducted in 7 general practices in Uppsala County, Sweden, where pharmacists were employed. Activities performed by pharmacists were self-reported during March 2021. Participant observations and semi-structured interviews with pharmacists were conducted between October and November 2021. Self-reported activities were categorised and analysed using descriptive statistics. Qualitative data were analysed using conventional content analysis.

**Results:**

In total, 174 activities were self-reported by 8 pharmacists. Two pharmacists were observed for 2 days each, and 6 pharmacists were interviewed. Their main task was conducting medication reviews in older patients with polypharmacy. In addition, they handled a broad variety of drug-related questions and treatment follow-up. Pharmacists described working in a more efficient and needs-based manner over time. They stressed that working at the practice increased their accessibility to and their collaborative work with other healthcare professionals, and enabled them to meet patients face-to-face. Future challenges include defining tasks more clearly, assuming greater responsibility for patient care, and meeting the growing demand for pharmacists in general practice.

**Conclusion:**

Pharmacists in general practice in Sweden perform a broad variety of tasks related to identifying, resolving and preventing drug-related problems, mainly in older patients with polypharmacy.

**Supplementary Information:**

The online version contains supplementary material available at 10.1007/s11096-023-01619-4.

## Impact statements

- This study provides a better understanding of the work of pharmacists in general practice, which supports further implementation and integration of general practice-based pharmacists.
- An overview of the broad variety of pharmacists’ drug-related tasks may facilitate definition and clarification of the scope of pharmacists’ practice in primary care.
- This study indicates that future research should focus on the development and implementation of advanced tasks in general practice, such as responsibility for the evaluation of treatment effects in certain patients.

## Introduction

The aging population and increased prevalence of multimorbidity result in the prescription of more medications and thereby complex pharmacotherapy [1]. This complexity may lead to individual therapies becoming inappropriate and even harmful for patients, putting pressure on healthcare systems [2]. At the same time, there are increasing shortages of physicians and nurses in many countries [3]. In primary care—central to the provision of health care and a gatekeeper to hospital care—general practitioners (GPs) are experiencing an increased workload, challenging the provision of high-quality care [4–6]. Pharmacists, who are qualified medication experts with a range of knowledge and skills, have been introduced in general practice to cope with this challenge [7, 8]. Evidence suggests that pharmacists can provide valuable services to ease the burden on general practice in addition to improving patient safety and health outcomes [9–11]. Some countries including Australia, Canada, the United Kingdom (UK) and the United States of America (USA), have well-established general practice-based pharmacists compared to other parts of the world. The pharmacists in these countries undertake a range of activities such as medication reviews and management of chronic conditions [11, 12]. In many European countries like Sweden, the concept of pharmacists in general practice is relatively new, and literature on their work is scarce. A lack of knowledge about what these pharmacists can contribute to general practice may hinder the development of the profession. A better understanding of pharmacists’ current tasks can promote further implementation of pharmacists in general practice, increasing their contribution to high-quality care and patient safety.

### Aim

The aim of this study was to explore the tasks performed by pharmacists in general practice in Sweden.

### Ethics approval

Ethical considerations were made, informed consent was obtained from all individual participants. All data were deidentified during analysis and reporting to ensure the participants’ anonymity. According to the Swedish Ethical Review Act [13], no ethical approval was required for this study.

## Method

### Study design

This study employed a qualitative case study approach that involved 3 data-collection methods: self-reporting tasks, participant observation and semi-structured interviews. A case study is an in-depth investigation of a phenomenon within a specific social system (a case) in its real-life context [14]. For this study, the case of pharmacists in general practice within Region Uppsala, Sweden, was investigated, with focus on the tasks that these pharmacists performed.

### Setting

Region Uppsala is steered by the local county government and responsibilities include health care of all 390,000 inhabitants in Uppsala County [15]. Approximately half of the general practices within the county are operated by the region. Since 2019, Region Uppsala has invested in permanent pharmacist positions within general practice to improve medication use and safety in primary care. The primary role of these pharmacists was to conduct medication reviews to identify, resolve and prevent drug-related problems in older patients using multiple medications (polypharmacy). Other tasks could be performed based on the needs and prerequisites of the specific workplace. For this study, all 7 general practices where pharmacists were working, either physically or remotely, within Region Uppsala were included. One of these practices, the geriatric outpatient unit, was for people aged over 65 years with chronic conditions and who had difficulties accessing their general practice. The other 6 practices were considered standard general practices serving between 8000 and 19,000 patients.

### Sampling and recruitment

The 9 pharmacists working at or for the general practices constituted the potential study population. For the self-reported tasks and interviews, all 8 pharmacists working in general practice within Region Uppsala during the data collection period were approached by e-mail and agreed to participate (one pharmacist being on parental leave during each part). Two pharmacists were recruited for participant observation. They were chosen because they were the only two pharmacists with at least 1 year experience working in general practice, were on location during the Covid-19 pandemic, and were not one of the authors. Additionally, we aimed to conduct a semi-structured interview with one pharmacist from each of the general practices, including those who had been observed.

### Data collection

Tasks were self-reported by pharmacists working throughout March 2021. They were instructed to report all performed tasks, on a daily basis, including requests from other healthcare professionals (HCPs), besides conducting medication reviews and excluding non-clinical work (e.g., participating in workplace meetings). The rationale for not including medication reviews was that we already knew that pharmacists perform this activity on a daily basis as their main task. For each task, the following information was reported in an online shared Microsoft Excel® spreadsheet, only accessible for the pharmacists: date, one-sentence description of the task and who initiated/requested the task.

Participant observation and semi-structured interviews were conducted between October and November 2021 by RK. Two pharmacists were observed during two full working days each. The observations were unstructured to allow an inductive approach. An observation protocol, adapted from a previous study [16], was used to record the tasks/activities the pharmacists performed, including when, how, where and with whom they interacted (Supplementary material). Apart from observations, the researcher also noted her own thoughts and interpretations, as well as information provided by the pharmacists during informal interviews, which was also considered relevant data for the purpose of this study.

For the semi-structured interviews, an interview guide was developed using Jacobs et al. [17]. The interview topics were based on the previous experience and knowledge of the researchers: the pharmacist’s tasks and work activities, patient population, collaboration with other HCPs, workplace and setting as well as perspectives on the future development of and challenges for pharmacists in general practice (Supplementary material). The interviews were conducted online through Zoom (a video conferencing system), audio-recorded and transcribed verbatim by the same researcher (RK).

### Data analysis

Data were analysed iteratively with conventional content analysis [18]. Self-reported tasks were analysed by the first author (TK) including familiarisation with the data, coding tasks and task groups. A second categorisation was performed according to the medications (drug group or drug treatment) involved in each task, if such data were available. The free text data about who had initiated or requested the task were also categorised. Finally, categories were analysed with descriptive statistics. Observation and interview data were thematically analysed by the same researcher (RK) who had collected the data along with one of the other researchers (SKS for observation data and TK for interview data). First, each pair of researchers independently read the data to perform initial coding. Then, a discussion was held between the two researchers about conflicting codes to reach consensus on a coding scheme and categorisation. RK then further categorised and (re)coded the data under the supervision of the other researchers. Afterwards, the third researcher (TK for observation data and SKS for interview data) critically reviewed the results. Finally, the results of the observations were incorporated in the themes and categories that emerged from the interviews, as all observations fitted into the interview themes and categories.

### Research team and trustworthiness

All observations and interviews were performed by a final-year pharmacy student without prior experience of qualitative research (RK). Before the study, she received an introduction to qualitative research (by SKS) and familiarised herself with the basic concepts of participant observation and semi-structured interviewing. SKS is a social scientist with extensive experience in qualitative research within pharmacy practice. TK is a pharmacist and assistant professor of pharmaceutical care. He worked as a pharmacist in general practice in Region Uppsala during data collection, hence participating in the self-reporting and in a semi-structured interview. He is trained and experienced in qualitative research. He was aware that being both researcher and participant had the potential risk of having a too positive view on the pharmacists’ work and mixing study data with personal experience, hence paying close attention to the reported findings being linked to the data.

A process of member checking by participating pharmacists was conducted in September 2022. All pharmacists working in general practice within Region Uppsala were sent a summary of the observation and interview findings and invited to provide reflections to verify the description of the tasks identified. SKS and TK held an online discussion with the pharmacists via Zoom, during which the pharmacists provided feedback on the study findings (Supplementary material). The study findings were adapted accordingly. An audit trail of participant recruitment, data collection and analysis was kept by RK to trace the course of the study and increase confirmability. The Standards for Reporting Qualitative Research were adhered to [19].

## Results

### Self-reported tasks

In total, 174 tasks were reported by 8 pharmacists (Table 1). Of these, 161 were requested by other health professionals: 124 (76%) by GPs, 26 (16%) by nurses and 13 (8%) by either an assistant nurse, dietician, general practice manager, medical secretary, midwife or patient. The most frequently reported task was drug-related requests or problems concerning an individual patient (n = 80, 46%), followed by questions about the electronic health record (EHR), the multidose drug dispensing (MDD; a dosing aid for patients with disposable bags containing the medications) system or about licences for non-registered drugs (n = 41, 24%; e.g., how to change a prescription to MDD).

**Table 1:** Pharmacists’ self-reported tasks (n = 174, reported by 8 pharmacists)

Self-reported tasks	Number of times reported (% of total)
**Manage drug-related request or problem concerning an individual patient**	**80 (46)**
Drug selection in patient with complex treatment	26 (15)
Adverse drug effect or intolerance	18 (10)
Drug administration	13 (7)
Dosage regimen or drug titration	10 (6)
Drug-drug interaction	7 (4)
Contra-indication	6 (3)
**Manage or answer a question about EHR/MDD systems or licences for non-registered drugs**	**41 (24)**
**Manage general drug-related request or problem**	** 30 (17)**
Availability or deregistration of product	7 (4)
Drug selection in patients with complex treatment	7 (4)
Drug cost	5 (3)
Adverse drug effect or intolerance	3 (2)
Contra-indication	1 (1)
Dosage regimen or drug titration	1 (1)
Drug-drug interaction	1 (1)
Not reported or unclear	5 (3)
**Provide information or education to other healthcare professional(s)**	**7 (4)**
**Provide support with developing or updating local routines**	**6 (3)**
**Perform drug preparation for administration to patient**	**4 (2)**
**Participate in multidisciplinary team discussion**	**3 (2)**
**Participate in pharmacotherapy quality improvement project**	**3 (2)**

*EHR* electronic health record, *MDD* multidose drug dispensing

In 106 cases, the drug group or drug treatment involved was reported (Table 2). Pharmacists described dealing with requests or problems related to all kinds of pharmacotherapy relevant to primary care. The most frequently involved drug group or treatment was antidepressants and anxiolytics (n = 16; 15%), followed by pain treatment (n = 12; 11%).

**Table 2:** Drug group or treatment involved in the self-reported tasks (n = 106)

Drug group or treatment	Number of times reported (% of total^a^)
Antidepressants and anxiolytics	16 (15)
Pain treatment	12 (11)
Vaccinations	9 (8)
Diabetes treatment	9 (8)
Asthma and COPD treatment	7 (7)
Gastrointestinal treatment (PPIs, laxatives and flatulence treatment)	6 (6)
Antihypertensives	6 (6)
Antibiotics and antimycotics	6 (6)
Lipid lowering drugs	5 (5)
Vitamins and iron supplements	3 (3)
Dermatologicals	3 (3)
Anticonvulsants	3 (3)
Sleep disorder treatment	3 (3)
Other cardiovascular drugs	2 (2)
Osteoporosis treatment	2 (2)
Oral antihistamines and corticosteroids	2 (2)
Obesity treatment	2 (2)
Other treatment (gout, hypothyroidism, incontinence and nausea)	4 (4)
Multiple treatments involved (multimorbidity or polypharmacy)	6 (6)

*COPD* chronic obstructive pulmonary disease, *PPI* proton pump inhibitor

^a^Number of times not reported or not applicable: 68 out of 174 (e.g. all technical or administrative questions about the electronic health record or multidose drug dispensing system)

Participant observation and semi-structured interviews The 2 pharmacists who were observed and interviewed had 2 and 13 years of clinical experience, respectively, in health care. One had obtained a post-graduate clinical pharmacy degree. Additionally, 4 pharmacists who worked in different general practices, including the geriatric outpatient unit, were interviewed (6 pharmacists in total, Table 3). These pharmacists had 5–20 years of clinical experience and one had obtained a post-graduate clinical pharmacy degree.

**Table 3:** Characteristics of the pharmacists participating in the observations and interviews (n = 6)

Characteristic	Value
Female gender, no. (%)	4 (66%)
Clinical experience, years, range	2–20
Clinical pharmacy post-graduate degree, no. (%)	2 (33%)

**Fig. 1:**
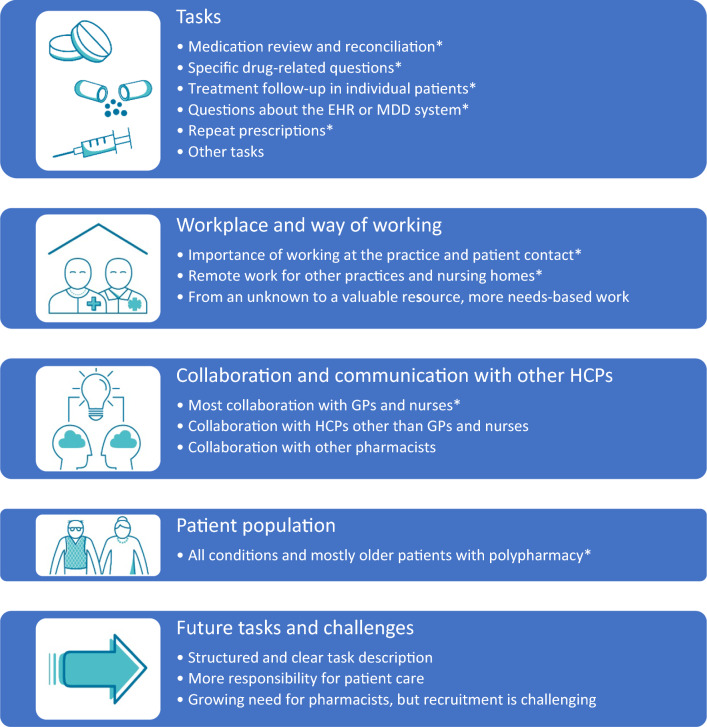
Five themes, each with 1 to 6 categories, concerning what pharmacists do in general practice. Findings from the participant observation (*) have been integrated in the themes that emerged from the semi-structured interviews. *Covid-19* coronavirus disease 2019, *EHR* electronic health record, *GP* general practitioner, *HCP* healthcare professional, *MDD* multidose drug dispensing

An example of a working day, based on the observations, is presented in Box 1. Other findings from the participant observation are incorporated in the 5 themes and multiple categories that emerged from the semi-structured interviews (Fig. 1). These themes and categories are described below, supported by illustrative quotations from the interviews (P1-6). No major changes were made after member checking; rather, the results were supported by the pharmacists.

**Box 1:** An example of a pharmacist’s typical working day in general practice, based on the observations

8:45 AM Pharmacist begins the day by checking the post-box and starting the computer to check the appointment schedule and messages in the electronic health record (EHR) system. Prepares for the first medication review and/or dealing with a drug-related question 9:00 AM Coffee break, mainly to meet and quickly catch up with other healthcare professionals (HCPs) who take a break at that time 9:15 AM Further preparation and accessing different information sources (e.g., medical history and laboratory results in the patient’s EHR or treatment guidelines) to deal with questions received through the EHR system and/or prior to patient contact. Writes answers to questions that can be handled directly through the EHR messaging system 9:45 AM Phone call to a patient who had a question about whether there are any interactions between prescribed medications and vitamin products that the patient is taking. Makes a note in the patient’s EHR to document what was discussed during the phone call 10:00 AM A patient visits the pharmacist for medication reconciliation as part of a medication review. After the reconciliation, the pharmacist walks to the general practitioner (GP)’s room to inform about the potential change in pharmacotherapy that was discussed with the patient. Documents the discussion with the GP, writes a preliminary note in the patient’s EHR, including the results of the medication review 11:00 AM A nurse knocks on the door and asks about which of a patient’s medications can be crushed, because the patient has difficulties swallowing certain tablets 11:15 AM Phone call to a patient to answer her/his question regarding possible side effects of a drug she/he is taking 11:45 AM Checks the e-mail and Microsoft Teams group to respond to questions from pharmacist colleagues 12:00 PM Lunch break including quickly discussing a patient case with another HCP and asking for clarification about a previous request. A GP asks the pharmacist to help with reporting an adverse drug effect to the Swedish medical product agency 0:45 PM Phone call to a patient to follow up on the (side) effects of antihypertensive treatment that had recently been changed 1:00 PM Logs into the web-based system to deal with requests for repeat prescriptions. Assesses and processes requests made by 12 patients. Walks to the nurses’ room to ask about how to process one specific request 2:00 PM Another patient visit in the context of a medication review, specifically addressing the patient’s inhaler technique. Pharmacist explains the difference between different inhalers and proposes substituting one inhaler for a more appropriate inhaler. After the patient visit, the pharmacist writes a message to the GP with a specific proposal regarding the substitution 2:30 PM Logs into the EHR referral system to deal with a request from a GP in a different practice for a medication review for a specific nursing home patient. The pharmacist performs the medication review based on the information received in the referral, the patient’s EHR and the multidose drug dispensing system. Writes pharmacotherapy recommendations in the patient’s EHR and as a response to the GP’s request 3:15 PM Finalises notes, sends messages in the EHR system that were not dealt with during the day and forwards the adverse drug effect report to the medical product agency 3:45 PM Pharmacist goes home

**Theme: tasks**

**Medication review and reconciliation**

Performing medication reviews was the pharmacists’ “*main job”* (P1), which was both observed and stated by the pharmacists. Often, the reason for performing a medication review was a specific DRP (e.g., a suspected adverse drug effect). Pharmacists also performed reviews prior to an appointment for a yearly check-up by the GP.

Medication reconciliation, the process of ensuring a correct medication list, was the first step of a medication review. This process was often combined with a discussion with the patient (or caregiver) about the indication(s) for drug treatment, how and when to take the medications, any DRPs (e.g., non-compliance issues) and any recommendations to optimize treatment. Medication reconciliation could also be requested and performed as a single task to reduce the GPs’ workload. For example, this was done by the pharmacist before the introduction of an MDD system or after hospital discharge. Some pharmacists were able to correct and prepare the patient’s medication list in the EHR for the GP to approve and prescribe.

We observed that both before and after medication reconciliation, pharmacists spent time assessing different information sources (e.g., medical history and laboratory results in the patient’s EHR or treatment guidelines) to thoroughly review the patient’s pharmacotherapy, find solutions for identified DRPs and optimize the patient’s treatment. Pharmacists also made notes in the EHR after each contact with a patient.

**Specific drug-related questions**

Many activities involved answering specific drug-related questions. According to the pharmacists, any drug-related question could be dealt with (see Table 4 for examples from the observations). Sometimes, questions were answered directly by the pharmacist, while other questions required the pharmacist to read the patient’s EHR, refer to other information sources or contact the patient.

**Table 4:** Examples of drug-related questions that the pharmacists received during the observations

Topic of question	Asked by
Dosage of an antidiabetic drug and its relation to muscle cramps and abdominal pain as potential side effects in an individual patient	GP
Differences between various brands of injectable drugs	Nurse
Alternative treatment for a drug that is not tolerated by an individual patient	GP
Differences between allopurinol and febuxostat as treatment options in gout	GP
A licence to prescribe a compound drug containing papaverine	GP
Equivalent dosage or product to compensate for specific drug shortages	GP
Interaction between apixaban and herbal medicinal products and vitamins	Nurse

*GP* general practitioner

**Treatment follow-up in individual patients**

During the observations, both pharmacists carried out follow-up phone calls with patients, e.g., to evaluate the effect after discontinuing a certain drug or to address treatment adherence. Most pharmacists also mentioned during the interviews that they frequently followed up with patients, either by telephone or in person. One pharmacist mentioned measuring blood pressure and ordering laboratory tests to support the follow-up process.

“*I take the initiative for some tasks, others I sort of get assigned by them [doctors and nurses] where it's booked in my schedule, like today I have to contact this patient who has increased his medication.*”—P6.

**Questions about the EHR or MDD system**

The pharmacists supported other HCPs when they had questions about drug-related digital systems or modules (i.e. the medication module in the EHR system and the MDD system). The pharmacists mentioned that GPs and nurses relied on the pharmacist’s expertise when initiating or making changes to the MDD system.

**Repeat prescriptions**

Three pharmacists (one of whom this task was observed) reduced the workload of nurses by performing some of their work regarding repeat prescriptions via a web-based portal or by telephone. Neither nurses, nor pharmacists are authorized to renew prescriptions, so an assessment was made by the pharmacist whether the renewal could be sent directly to the GP to sign or if it required a further GP assessment.

“*On Mondays […] there is usually a lot for the nurses to take care of […] so I generally step in and reduce the load of prescription renewals as much as I can*.”—P2.

**Other tasks**

Pharmacists mentioned updating and educating other HCPs about drug-related news or changes in local guidelines. Some had also supported the development of medication management routines at the general practice.

**Theme: workplace and way of working**

**Importance of working at the practice and patient contact**

One of the observed pharmacists had a separate room within the general practice, while the other relied on a spare GP room being available. Most pharmacists mentioned the importance of working at the practice, which enhanced their work in different ways: increased collaborative work with other HCPs, having face-to-face patient contact and being able to join multidisciplinary meetings and rounds (e.g., diabetes rounds with a GP and nurse).

“*I had a room next to the reception and between different doctors, so everyone could find me. Generally, I was there three days a week, those were fixed days, so they knew which days I was there, otherwise it wouldn't work*.”—P5.

Contact with patients was preferred in person at the practice, but could also be out over telephone. For nursing home residents and geriatric outpatients, a validated form to identify possible drug-related symptoms was often filled in by the carer with or on behalf of the patient. This provided a basis for medication reconciliation, combined with data from the EHR and MDD system. Although it was considered *“valuable to see when patients […] show their medications at home”* (P4)*,* pharmacists rarely visited patients at home, because it was *“time consuming”* (P2).

**Remote work for other practices and nursing homes**

Referrals for medication reviews or other requests could be sent by HCPs from all general practices in the region through the EHR system. These requests were then handled through the same system.

“*So, what I do here is that I work with the patients in the geriatric outpatient unit and then I respond to referrals from GPs from across the region*.”—P3.

**From an unknown to a valuable resource, more needs-based work**

When pharmacists started working at their practice, much time was spent on actively seeking out patients to invite for medication reviews. Pharmacists explained that they had become more efficient and valuable over time, working in a more needs-based manner as HCPs started approaching the pharmacists with all kinds of requests.

“*They have started understanding what you can use a pharmacist for, but it takes some time for them to feel comfortable asking all types of questions*.”—P6.

**Theme: collaboration and communication with other HCPs**

**Most collaboration with GPs and nurses**

As mentioned by the pharmacists and as observed, they mostly collaborated with the GPs and nurses *“obviously […] because they work closest to medications”* (P3). Requests were made through the EHR messaging system, written notes or in person. When working at the general practice, pharmacists, GPs and nurses could easily discuss patient cases and pharmacists would provide pharmacotherapy recommendations or answer questions.﻿

**Collaboration with HCPs other than GPs and nurses**

Dieticians, physiotherapists, psychologists and social workers were most often mentioned by the pharmacists as other HCPs, apart from GPs, with whom they collaborated.

“*I would say that there is no [profession] that I don't work with, but I work with some more than others*.”—P6.

**Collaboration with other pharmacists**

The general practice pharmacists could contact community pharmacists about prescription errors in individual patients or in the event of drug shortages. However, they mainly consulted their colleagues in primary or hospital care to discuss patient cases. The pharmacists in primary care felt like a group that supported each other.

“*We have a great chat group […] and it is worth its weight in gold. […] You receive excellent help and quick answers*.”—P4.

**Theme: patient population**

**All conditions and mostly older patients with polypharmacy**

Pharmacists managed *“all possible clinical conditions”* (P3) treated in primary care. Two of them mentioned being more specialised: one in inhaler therapy for chronic lung disease patients and one in patients using oral anticoagulant treatment. Although pharmacists dealt with patients of all ages, they particularly focused on older patients in relation to polypharmacy.

“*We have decided that all patients admitted to the geriatric outpatient unit and all patients who come for yearly check-ups should receive a medication review*.”—P2.

**Theme: future tasks and challenges**

**Structured and clear task description**

According to the pharmacists, the task description for pharmacists in general practice seemed unclear both to themselves and to others. Pharmacists stated the need for a clearer definition of tasks, enabling a more effective way of working. They wanted decisions about which tasks to prioritize to be made at leadership or policy level. At the same time, specific tasks should be adapted locally because *“different general practices have different needs”* (P4).

“*It's time to define which patients, which type of questions you are to deal with so that it's clear to others*.”—P5.

**More responsibility for patient care**

In order to work more effectively, most pharmacists wanted more responsibility and autonomy regarding patients’ treatment. For example, responsibility for evaluating the effects after the start or change of pharmacotherapy. The pharmacists stated that gaining the right to prescribe in certain situations would make this possible in the future, but would need to be introduced gradually and carefully.

“*I believe that we can relieve the doctors of many tasks in the future, but it must happen gradually so that they are on board*.”—P4.

**Growing need for pharmacists, but recruitment is challenging**

Pharmacists identified lack of time and resources as two of the main challenges in their work. With an increased understanding of what pharmacists can contribute to, the need for pharmacists and their services seems to be growing and becoming *“inexhaustible”* (P6).

“*Right now, our challenge is that there should be more of us, it's difficult to recruit experienced pharmacists, the demand is increasing rapidly in primary care*.”—P3.

## Discussion

This study employed a case study approach with data generated from self-reported tasks, participant observations and semi-structured interviews to investigate what tasks pharmacists perform in general practice in Region Uppsala, Sweden. In addition to conducting medication reviews for older patients with polypharmacy, pharmacists handled a broad variety of drug-related requests and problems for which they mainly collaborated with GPs and nurses.

Similar to the findings from two literature reviews with mainly studies from the UK, USA, Australia and Canada [11, 12], and a recent study in Australia [20], pharmacists in Region Uppsala mostly performed tasks related to medication management (e.g., medication reviews, DRPs and repeat prescriptions), hence were involved in detecting, resolving and preventing DRPs. In countries where pharmacists can prescribe medications, medication management also included prescribing activities [11, 12], which was an option carefully considered for the future by some pharmacists in the current study. Other activities were related to collaboration with various HCPs, serving as a liaison between general practice and community pharmacy, and counselling and educating patients and other HCPs [11, 12].

Pharmacists in Region Uppsala performed fewer tasks related to patient examination and screening (e.g., ordering and reviewing laboratory tests and physical examination), chronic disease management (e.g., formulating and following-up on care plans for patients) compared to reports in other studies [11, 12, 20]. These activities require a higher degree of autonomy and responsibility for patient care. Similar to previous studies in Canada [21, 22], pharmacists in the present study expressed a desire to perform such tasks in the future, and assume greater responsibility for patient care. Systematic reviews on the effects of pharmacist services in general practice appear to report more favourable results in studies involving pharmacists in the follow-up of patients, rather than delivering medication reviews, education or drug information [9, 10].

Activities related to auditing and quality assurance are commonly reported by general practice pharmacists in different countries [11, 12, 20]. In this study, these activities were rarely performed but mentioned as potential tasks. Pharmacists stressed that it would be impossible to perform all these activities, as there were too few pharmacists to meet the increasing demand in general practice. Providing a clearer task description may enhance pharmacists’ contribution in general practice, as successful multi-professional collaboration and teamwork are dependent on understanding each other’s roles [23, 24]. At the same time, general practices have different needs, hence efficient use of pharmacists will likely include performing a variety of tasks [7]. The pharmacists in the present study felt that their work became more efficient and valuable over time, as other HCPs understood what pharmacists could contribute to. They adapted their work to local needs, hence becoming more integrated in the general practice. The presence of a pharmacist in the practice is essential for such local work adaptation and successful collaboration with other HCPs [7, 25, 26].

### Methodological strengths and limitations

Research trustworthiness and rigour [27] were addressed in diverse ways throughout the research process (Table 5). Some limitations also need to be considered. First, the number of pharmacists observed (n = 2) and interviewed (n = 6) is limited and interview transcripts were neither checked for accuracy nor returned to the participants for comments. However, all pharmacists working in general practice in Region Uppsala participated in this study and preliminary results were member checked. Second, data collection could have been influenced by the pharmacy-background of two of the researchers (TK and RK) and the participants themselves, possibly presenting a more positive description of pharmacists’ performance. This potential risk was mitigated by the involvement of a third researcher (SKS) with a social science background who scrutinized all study findings. In addition, the researchers chose to acknowledge their predispositions and tried to describe the tasks as reported and observed, leaving judgement of their value and relevance to the reader. Third, the study was carried out during the Covid-19 pandemic, which reduced the number of pharmacists working in general practice, possibly having a negative impact on their tasks, for example having fewer face-to-face patient contact.

**Table 5:** Strategies to ensure the trustworthiness and rigour of the research process [27] employed in this study

Credibility	Adoption of appropriate and established research methods [14] Sampling of all pharmacists as participants (Sampling and recruitment) Triangulation of findings through different data collection methods (Data collection) Use of different researchers to independently analyse data (Data analysis) Familiarity with the study context among the researchers (Research team and trustworthiness) Description of researchers’ background and training (Research team and trustworthiness) Member checking by participating pharmacists (Research team and trustworthiness) Examination of previous research to frame findings (Discussion)
Transferability	Description of the context (Study design and Setting) and comprehensive description of the findings (Results)
Dependability	Comprehensive methodological description to enable the study to be repeated (Methods) Reflective appraisal of the study (Discussion)
Confirmability	Recognition of limitations in the study methods and their potential effects (Discussion) Audit trail of the recruitment, data generation and analysis (Data collection and Data analysis)

### Interpretation and implications

This study provides a better understanding of what pharmacists do in general practice, showing a broad variety of drug-related tasks, and thus supporting further implementation and integration of general practice-based pharmacists. Involving relevant stakeholders (e.g., other HCPs, patients and policy makers) in this process may result in a better comprehension of and more support for the pharmacist’s role in general practice. To further enhance the pharmacists’ contribution in patient care, decisions should also be made at policy or practice level about tasks that require a higher degree of autonomy and responsibility. Future research may focus on the development, implementation and evaluation of such advanced tasks in general practice that include patient examination and screening as well as formal chronic disease management.

## Conclusion

Pharmacists in general practice in Sweden perform a broad variety of tasks related to identifying, resolving and preventing drug-related problems, mainly in older patients with polypharmacy.

### Supplementary Information

Below is the link to the electronic supplementary material.

Supplementary file1 (DOCX 92 kb)
