# Supplementary material for: Pharmacists in general practice: what do they do? A qualitative case study
Source: Int J Clin Pharm. 2023 Aug 12;45(6):1472–82. doi: 10.1007/s11096-023-01619-4 (PMC10682112; doi:10.1007/s11096-023-01619-4)
Supplement: Supplementary file 1 — Supplementary file1 (DOCX 92 kb) [file 11096_2023_1619_MOESM1_ESM.docx]

**Observation protocol**

| **Practice name** | **Manager’s name** | **Pharmacist’s name** | **Observer’s name** | **Date** |
| --- | --- | --- | --- | --- |
| **Start of observation (time):** | | **End of observation (time):** | | |
| Part 1: Everything observed should be documented in the field notes.  Part 2: Personal feelings, opinions and reflections/interpretations should be recorded separately.   \| Part 1 \| Part 2 \| \| --- \| --- \| | | | | |

**Interview guide**

**Introduction to the interview**

Hello [the name of the person being interviewed]. I am Rouzi Koumi, a pharmacy student in my ninth term at Uppsala University. I have chosen to perform a social pharmacy research project and am interested in deepening my knowledge of the role of a clinical pharmacist in primary care. Therefore, I wish to interview you. I would first like to obtain your consent for the interview [hand over the consent form]. On consent to take part in the interview, ask if the person being interviewed has any questions. Then start the audio recording and begin the interview.

Background information:

1. What level of education do you have?
2. How long have you worked as a pharmacist and a clinical pharmacist?
3. Where have you worked as a pharmacist?
4. Have you worked as a clinical pharmacist in hospital care before starting in primary care? How long have you worked in your current position?

Interview questions:

- Can you tell me what your working day looks like, what tasks you perform?
  Are there any specific tasks that you perform more frequently?

*It is important to ask more specific follow-up questions such as: when, how, in what situations?*

- Do you work on-site/remotely? What is it like?
  If on-site, do you have your own room, or do you share a room with other healthcare professionals? Do you perform your tasks in the healthcare centre where you work, or do you make home healthcare visits, for example?
- Which patients do you take care of?
  Are the patients of a specific age group? Specific clinical conditions? Or do your tasks cover all types of clinical condition?
- Which healthcare professionals do you collaborate or come into contact with?
  For what reason and in which situations do you have contact with other professionals?
- How have your tasks changed since you started?
- How to further educate yourself – who to contact if you need a "second opinion"?
- What is your view about the future of pharmacists in primary care?
  What do they contribute? How can their role be developed? What challenges do you see now and in the future?

What to say at the end of the interview:

We have now reached the end of our interview. Is there anything we have not talked about that you would like to add?
I want to thank you for this interview, for your time and participation. Can I contact you again if I need any additional information? May I ask if you would like to receive the results of my research project when it is completed?

**Member checking discussion guide**

1. To what extent is the summary of the tasks you (plural form in Swedish) perform and experience in your work accurate, both in relation to the period October-November 2021 and in the current situation?
2. Are there results in the summary that are not correct? If so, what?
3. Are there tasks that were done in 2021 (when the data was collected) that are no longer being done?
4. Are there tasks that you have started to do (more or differently) since the study's data was collected?
5. How do you reason about the categories and their explanation under the theme "Future tasks and challenges"?
